# Cholinergic Machinery as Relevant Target in Acute Lymphoblastic T Leukemia

**DOI:** 10.3389/fphar.2016.00290

**Published:** 2016-08-31

**Authors:** Oxana Dobrovinskaya, Georgina Valencia-Cruz, Luis Castro-Sánchez, Edgar O. Bonales-Alatorre, Liliana Liñan-Rico, Igor Pottosin

**Affiliations:** ^1^Centro Universitario de Investigaciones Biomédicas, Universidad de ColimaColima, México; ^2^Consejo Nacional de Ciencia y TecnologíaMéxico City, México

**Keywords:** acetylcholine, acetylcholinesterase, calcium signaling, choline acetyltransferase, muscarinic receptor non-neuronal cholinergic system, T leukemia, T lymphocyte

## Abstract

Various types of non-neuronal cells, including tumors, are able to produce acetylcholine (ACh), which acts as an autocrine/paracrine growth factor. T lymphocytes represent a key component of the non-neuronal cholinergic system. T cells-derived ACh is involved in a stimulation of their activation and proliferation, and acts as a regulator of immune response. The aim of the present work was to summarize the data about components of cholinergic machinery in T lymphocytes, with an emphasis on the comparison of healthy and leukemic T cells. Cell lines derived from acute lymphoblastic leukemias of T lineage (T-ALL) were found to produce a considerably higher amount of ACh than healthy T lymphocytes. Additionally, ACh produced by T-ALL is not efficiently hydrolyzed, because acetylcholinesterase (AChE) activity is drastically decreased in these cells. Up-regulation of muscarinic ACh receptors was also demonstrated at expression and functional level, whereas nicotinic ACh receptors seem to play a less important role and not form functional channels in cells derived from T-ALL. We hypothesized that ACh over-produced in T-ALL may act as an autocrine growth factor and play an important role in leukemic clonal expansion through shaping of intracellular Ca^2+^ signals. We suggest that cholinergic machinery may be attractive targets for new drugs against T-ALL. Specifically, testing of high affinity antagonists of muscarinic ACh receptors as well as antagomiRs, which interfere with miRNAs involved in the suppression of AChE expression, may be the first choice options.

## Introduction

Acute lymphoblastic leukemia is the most common type of cancer in infants and a leading cause of cancer-related deaths in children (Ward et al., 2014). Although T-ALL is 5–6 times less common than B-ALL, it is characterized by a worse prognosis, especially dismal in case of a relapse (Nguyen et al., 2008). Obviously, search for new efficient therapies for T-ALL needs to be continued.

Traditionally, the cholinergic system was considered specific to nerve cells that produce ACh and use it as a neurotransmitter. However, it is widely accepted nowadays that ACh, as a functionally important compound, evolutionarily predates the development of a nervous system. ACh plays important autocrine and paracrine functions in physiology of many types of non-neuronal cells. Accordingly, the concept of a “NNCS” is widely used (Kawashima and Fujii, 2003). Particularly, ACh was shown to be involved in proliferation, apoptosis, and cellular migration. Noteworthy, all these processes are fundamental for tumorigenesis, and there is growing evidence that the cholinergic signaling is an essential component in pathogenesis of various malignancies (Shah et al., 2009).

A variety of cellular types in the immune system, such as T lymphocytes, granulocytes, macrophages, mastocytes, and DCs have been reported to express the ACh-synthesizing enzyme ChAT; all immune cells, including T and B cells, macrophages and DC, express several subtypes of AChR (Kawashima and Fujii, 2000, 2003, 2004; Kawashima et al., 2015). However, the patterns of cholinergic elements vary considerably among different lymphocyte lineages and leukemic cell lines (Kawashima and Fujii, 2000, 2004; Fujii et al., 2008). There is accumulated evidence for the involvement of the cholinergic pathway in modulation of the immune response, by means of its effect on lymphocyte proliferation and differentiation, cytokine production, antigen presentation, and inflammation (Ofek and Soreq, 2013).

T cells are considered to be the main source of the ACh in blood, and they were proposed to represent an independent cholinergic system (Kawashima et al., 2012). There is a vast array of relevant data obtained on human MNLs, isolated T cells and leukemic cell lines derived from T-ALL. It was demonstrated that the ACh pathway represents an essential segment in calcium (Ca^2+^) signaling of T cells. At the same time, T-ALL pathogenesis is associated with an aberrant Ca^2+^ signaling (Bhojwani et al., 2015; Dobrovinskaya et al., 2015). Yet, to our best knowledge, no systematic attempts have been undertaken to link T lymphocyte cholinergic system with T leukemogenesis. In the present paper we will focus on the comparison of cholinergic system in healthy and leukemic T cells, to reveal the potential of this pathway as a pharmacological target in T cell derived neoplasias.

## T Cells As Important Components of Non-Neuronal Cholinergic System

As ACh is a short-lived compound, which is rapidly degraded by AChE and BChE, its presence in considerable amounts in blood of mammals argued for its origin from blood cells rather than from neurons. Indeed, about 60% of blood ACh originates from lymphocytes, and T lymphocytes are considered authentically cholinergic (reviewed in Kawashima and Fujii, 2000, 2004). Further in this review, the expression patterns of cholinergic elements in different lymphoid lineages and cell lines will be considered.

### Choline Uptake

Choline is a precursor for the ACh synthesis, as well for the synthesis of major membrane phospholipids, phosphatidylcholine, and sphingomyelin. Accordingly, any cell possesses the machinery for choline transport. Respective transporters belong to three distinct families: (1) CHT1; (2) CTL1-5; (3) polyspecific OCT with a low affinity for choline (Michel et al., 2006). Since the rate limiting step in the ACh synthesis is the uptake of choline, expression of choline transporters of high affinity seems to be required for effective ACh production. In nerve terminals, choline is exclusively taken up by CHT1 (Kuhar and Murrin, 1978). Although no data are available for isolated healthy human lymphocytes, robust presence of CTL1 protein in human spleen, as shown by Western blot, is indicative that normal T cells may express CTL1(Michel et al., 2006). Functional expression of high affinity CHT1 was demonstrated in leukemic cell line MOLT-3 (Fujii et al., 2003), which is characterized by extremely high ACh content (∼250 pmol/10^6^ cells) as compared to the leukemic lines CEM and Jurkat, ∼12.6 and 8 pmol/10^6^ cells, respectively (Kawashima and Fujii, 2004). Notably, CEM and Jurkat T cells did not express CHT1 (Fujii et al., 2003). Jurkat cells were reported recently to express intermediate-affinity CTL1 (Inazu, 2014). Thus, an elevated level of the ACh production in leukemias may be related to the expression of the CHT1.

### Acetylcholine Synthesis

Cytoplasmic ChAT is the primary enzyme that catalyzes both neuronal and non-neuronal ACh production from choline and acetyl coenzyme A. The presence of ChAT mRNA and protein has been detected in human and murine MNL, rat lymphocytes, human leukemic T cell lines, but, importantly, not in B cells (Fujii et al., 1995, 1998, 1999; Rinner et al., 1998; Kawashima et al., 2007). Unstimulated human CD4^+^, but not CD8^+^ T cells, express ChAT mRNA (Fujii et al., 1999).

Mitogenic stimulation with PHA potentiated activity of ChAT, ACh synthesis and release in human MNL and leukemic T cell lines, implicating the involvement of the cholinergic machinery in the regulation of the T cell activation and proliferation (Fujii et al., 1996, 1998, 2012a,b; Kawashima and Fujii, 2000). PHA is known to activate T cells through up-regulation of PLC-mediated production of the inositol-1, 4, 5-trisphosphate (IP_3_), followed by intracellular Ca^2+^ rise, and, in turn activation of Ca^2+^-dependent phosphatase calceneurin (Cn) and cascades of PKC and MAPK (Lichtman et al., 1983). A non-specific PKC activator PMA and the PKA activator dibutyryl cAMP were shown to up-regulate the ChAT activity, ACh synthesis and release from T leukemic cells MOLT-3. Cn inhibitor FK506 suppressed the PHA-induced ChAT mRNA expression, suggesting that Ca^2+^/Cn-mediated pathway is involved in the process of ACh synthesis in MOLT-3 cells (Fujii et al., 2012b).

Stimulation of T cells with ICAM-1, an intercellular adhesion molecule binding to the integrin CD11a, also up-regulates ChAT expression and ACh synthesis in T cells. Similar results were obtained with monoclonal antibodies against CD11a. Thus, the interaction of T cells with the activated vascular endothelium or other immune cells, such as macrophages, up-regulates the lymphocytic cholinergic activity (Kawashima et al., 2012).

Notably, as demonstrated in a rat model, T cells express ChAT and produce ACh from the early stages of maturation in thymus. It was suggested that ACh may serve as a mediator between maturating thymocytes and thymic stroma, and regulate apoptosis during positive and negative selection. Furthermore, ACh production increased after the mitogenic stimulation of isolated thymocytes *in vitro* (Rinner et al., 1999).

### Acetylcholine Release

In cholinergic neurons, ACh is synthesized in the cytosol and then transported into synaptic vesicles by VAChT, where it remains stored until a specific stimulation takes place (Varoqui and Erickson, 1996). ACh release from the cholinergic nerve terminals may be mediated by the exocytosis, evoked by cytosolic Ca^2+^ increase caused by membrane depolarization during an action potential, or alternatively, by synaptosomal membrane protein mediatophore, which translocates ACh in response to Ca^2+^ challenge (Israël et al., 1986, 1994, 1998; Cavalli et al., 1991; Malo and Israël, 2003; Dunant et al., 2009).

A non-neuronal VAChT was described in human SCLC, where a specific VAChT inhibitor vesamicol notably attenuated ACh release and cell proliferation (Song et al., 2003). However, there is no evidence for the presence of VAChT mRNA in human healthy lymphocytes or leukemic cell lines. It was proposed that, in contrast to neurons, lymphocytes synthesize and liberate ACh without storage (Fujii et al., 2008). Some plasma membrane proteins were shown to facilitate ACh liberation by translocation, like synaptosomal mediatophore mentioned above (Israël et al., 1986, 1994, 1998; Cavalli et al., 1991; Malo and Israël, 2003; Dunant et al., 2009) or OCT in human placenta and urothelium (Wessler et al., 2001; Kummer et al., 2006). In leukemic T cell lines CEM and MOLT-3, the presence of mediatophore, but not of OCT mRNA was shown. Activation with PHA significantly up-regulates ChAT and mediatophore expression in these cells, with a subsequent synthesis and release of ACh (Fujii et al., 2012a). Since meditophore releases ACh in a Ca^2+^- dependent manner (Dunant et al., 2009), one can suggest that physiological events, which provoke intracellular Ca^2+^ rise in lymphocytes (antigen activation and clonal expansion) may intensify the ACh release. However, this hypothesis needs to be proved experimentally. To date, there is no available information regarding mediatophore involvement in the ACh release by T cells derived from healthy donors.

### AChE Expression

Signaling events, mediated by ACh, are terminated, when ACh is hydrolyzed by the AChE and by a less specific BChE. Hence, to be able to regulate ACh level in nearby microenvironment, immune cells themselves should express ACh degrading enzymes. Indeed, classical studies undertaken with normal human peripheral blood lymphocytes demonstrated that T but not B cells populations possess high AChE enzymatic activity (Szelényi et al., 1982). The enzyme was shown to be membrane-bound and present in homogenous dimeric form (Bartha et al., 1987). More recently, expression of three different types of the AChE mRNAs has been detected in leukemic cell lines of T (CEM) and B (Daudi) lineages as well as in human peripheral blood lymphocytes (Tayebati et al., 2002).

Up-regulation of the AChE by PHA was reported in T cells (Szelényi et al., 1982; Kawashima et al., 2015). Accordingly, T cells activation is accompanied not only by an increased ACh synthesis and up-regulation of AChR expression, but also by the activation of their own ACh degradation mechanism. These findings further support the idea that T cells possess an independent cholinergic machinery.

Detailed studied carried out on 56 leukemic cell lines have demonstrated that AChE activity was significantly lower in cell lines derived from patients with T-ALL, when compared with cell lines originated from adult T cell leukemia (Rubinstein et al., 1984). Accordingly, the authors suggested that AChE activity may represent a maturation feature of T cells.

### Cholinergic Receptors and the Regulation of Immune Function

In accord with a binding affinity for two naturally occurred substances, muscarine or nicotine, cholinergic receptors are classified into two categories, muscarinic (mAChR) or nicotinic (nAChR), respectively, which possess no structural similarity one with another, although both could be activated by ACh.

Muscarinic belong to a class I subfamily of G-protein coupled receptors with seven conserved transmembrane domains and are represented by five distinct subtypes, M1-5 (Caulfield and Birdsall, 1998; Ishii and Kurachi, 2006). M1, M3, and M5 receptors are preferentially coupled to the Gq/11 subunit of G-proteins, with resulting stimulation of PLC-mediated production of the IP_3_ and subsequent increase of the intracellular Ca^2+^ concentration (Nahorski et al., 1997). In contrast, M2 and M4 are coupled mainly to G proteins of the Gi/o classes, typically leading to the adenylate cyclase inhibition and activation of inward-rectifier potassium channels (Yamada et al., 1998; Volpicelli and Levey, 2004). mAChR subtypes can regulate a wide network of signaling intermediates, including small GTPase Rho, phospholipase D, phosphoinositide-3 kinase, non-receptor kinases, and MAPKs (Kozma et al., 1997; Schmidt et al., 1997; Firth and Jones, 2001; Linseman et al., 2001).

Nicotinic nAChRs are pentameric complexes consisting either of α (1-10) subunits, homomeric (in case of α7 and α9) or heteromeric ones, or of a combination of α and β subunits (in muscle tissue δ, ε, and γ subunits may be present as well). In neurons and muscle nAChRs form a ligand (ACh and nicotine)- gated cation channels. Depending on the subunit composition, these channels display a different sensitivity to agonists and a different degree of Ca^2+^/Na^+^ selectivity, with homomeric α7 complex being the most Ca^2+^-selective and less nicotine-sensitive (Russo et al., 2014; Zhao, 2016 and references therein).

Most immune cells express several subtypes of both mAChR and nAChR. The presence of AChR in normal rodent and human lymphocytes was confirmed in numerous binding studies using radiolabeled ACh, nicotine and specific muscarinic or nicotinic antagonists (reviewed in Kawashima and Fujii, 2000). Such radioligands, however, are not selective for special receptor subtypes. The expression of mRNA encoding AChR subtypes in human MNL from healthy donors was analyzed by Sato et al. (1999). Although, the presence of at least some mAChR and nAChR subtypes was detected in all samples, the expression pattern varied significantly among individuals. M4 and M5 mAChR, and nAChR subunits α2, α5, and α7 were present in all samples (7/7); in some samples were also present M1 (5 of 7), M2 (5 of 7), M3 (5 of 7), α6 (2 of 7), β2 (3 of 7). These data evidenced the diversity of cholinergic system in human immune cells, where each person may respond to ACh stimulation in a different way. It was proposed that the expression pattern may be genetically determined or regulated by some factors such as infection and physiological stress (Kawashima and Fujii, 2000). It was found that levels of α5 and α7 nAChR subunits expression were significantly decreased in peripheral MNL from smokers, when compared with those from non-smokers (Fujii et al., 2008).

Immunohistochemical analysis and reverse transcription-polymerase chain reaction (RT-PCR) of MNL has shown the presence of several α and β isoforms like α3, α4, α5, α7, α9, α10, β1, and β2 (Hiemke et al., 1996; Lustig et al., 2001; Peng et al., 2004; Wongsriraksa et al., 2009). In isolated T cells populations, the expression of α2, α5, α6, α7, α9, α10, and β2 isoforms was revealed at mRNA and protein levels by RT-PCR and immunohistochemical analysis, respectively (Toyabe et al., 1997; Lustig et al., 2001). Caution should be paid to results on the occurrence of specific α-nAChR subunits, obtained exclusively by means of commercial antibodies. The latter, as shown in mice with a null mutation for a single nAChR subunit, also label collateral targets, with a level of immunoreactivity very similar to wild type (Moser et al., 2007). It was recommended, therefore, to employ alternative strategies and use, for instance, conjugated specific antagonists, like α -bungarotoxin (α -BTX; Jones and Wonnacott, 2005).

Reverse transcription-polymerase chain reaction analyses of nAChR in thymocytes subsets and T cells indicated that during the process of thymocyte maturation there is a down-regulation of α2 and α5, denoting their possible role in T cell development (Kawashima et al., 2012). Plasticity of the T-cell cholinergic receptors during differentiation was also demonstrated in murine model (Qian et al., 2011).

It is well-known that nAChR form ligand (ACh and nicotine)- gated cation channels in neurons and muscles. To better understand the physiological significance of these receptors in lymphocytes, single-cell records were attempted on both B and T subsets using standard patch-clamp techniques (Peng et al., 2004). However, lymphocytes recordings did not show any current response to a stimulation with ACh, nicotine or cholinergic receptor agonist carbachol. Then, nAChR may have a different mode of action in lymphocytes (Peng et al., 2004).

Non-channel function of α7 nAChR in human lymphocytes seems to be caused by partial duplication of the gene CHRNA7 coding for α7. Resulting new gene, human – specific CHRFAM7A, codes α7 duplicate nicotinic AChR- related protein (dupα7). Dupα7 assembles with α7 subunits (α7dupα7), but does not exhibit ACh or nicotine binding sites (Villiger et al., 2002). Expression of α7 and dupα7 in a 1:1 molar ratio in oocytes resulted in a decrease of ACh-stimulated current by more than 30%, suggesting that the CHRFAM7A gene product (dupα7) is a dominant-negative regulator of α7 ion channel function (Araud et al., 2011; De Lucas-Cerrillo et al., 2011). Human peripheral blood lymphocytes were shown to express large amounts of dupα7/CHRFAM7A, but little to no α7/CHRNA7 mRNA (Costantini et al., 2015a,b). High level of CHRFAM7A mRNA expression was detected also in purified population of human CD4^+^ T-lymphocytes (Rozycka et al., 2013). It was hypothesized that CHRFAM7A play significant role in human leukocyte biology. Specifically, the ratio of CHRFAM7A and CHRNA7 expression may be important in defining the human α7 function (Costantini et al., 2015a,b). As the CHRFAM7A gene is human-specific, the findings on α7 nAChR expression and function obtained in animal models should be taken with precaution and cannot be extrapolated to humans.

Although the underlying mechanisms are not well-understood, there is little doubt about the functionality of activated AChR in lymphocytes. Upon stimulation of healthy T cells with Con A, PHA, or nicotine the expression of α7 was up-regulated (De Rosa et al., 2005, 2009; Razani-Boroujerdi et al., 2007; Landais et al., 2010). ACh and muscarinic agonists stimulated DNA and RNA synthesis, IP_3_ accumulation, and lymphocyte proliferation in humans and rodents (reviewed in Kawashima and Fujii, 2000). Nicotine regulates cell proliferation, differentiation, migration, cell–cell interactions, and apoptosis (De Rosa et al., 2005, 2009; Liu et al., 2014). ACh and synthetic mAChR agonists insensitive to AChE hydrolysis, as well as muscarinic antagonists, were used to demonstrate that M3 and/or M5 stimulation induces transient intracellular Ca^2+^ rise, followed by extracellular Ca^2+^-dependent oscillations and up-regulation of the c-fos expression in T and B cells (Fujii and Kawashima, 2000a). Using AChR knockout mice, it was found that the M1/M5 pathway up-regulates IgG1 and pro-inflammatory cytokine production, while the α7 nAChR linked signaling has the opposite effect (Fujii et al., 2007; Kawashima et al., 2015). CD8^+^ T cells from M1-deficient mice show a defect in their ability to differentiate into cytolytic T lymphocytes (Zimring et al., 2005). Since muscarinic agonist Oxo-M stimulates the production of interleukin-2 (IL-2) and up-regulates the expression of IL-2 receptors (IL-2R) in PHA-stimulated T cells, it was proposed that ACh acts as an autocrine factor (reviewed in Kawashima and Fujii, 2003). It was postulated that the T-cell derived ACh, functioning via autocrine/paracrine pathways may be involved in the regulation of immune function (reviewed in Kawashima et al., 2015). However, there is as yet no *in vivo* direct evidence demonstrating that T cell- derived ACh binds to AChR on the same cell or neighboring cells, to exert this modulating effect.

**FIGURE 1:**
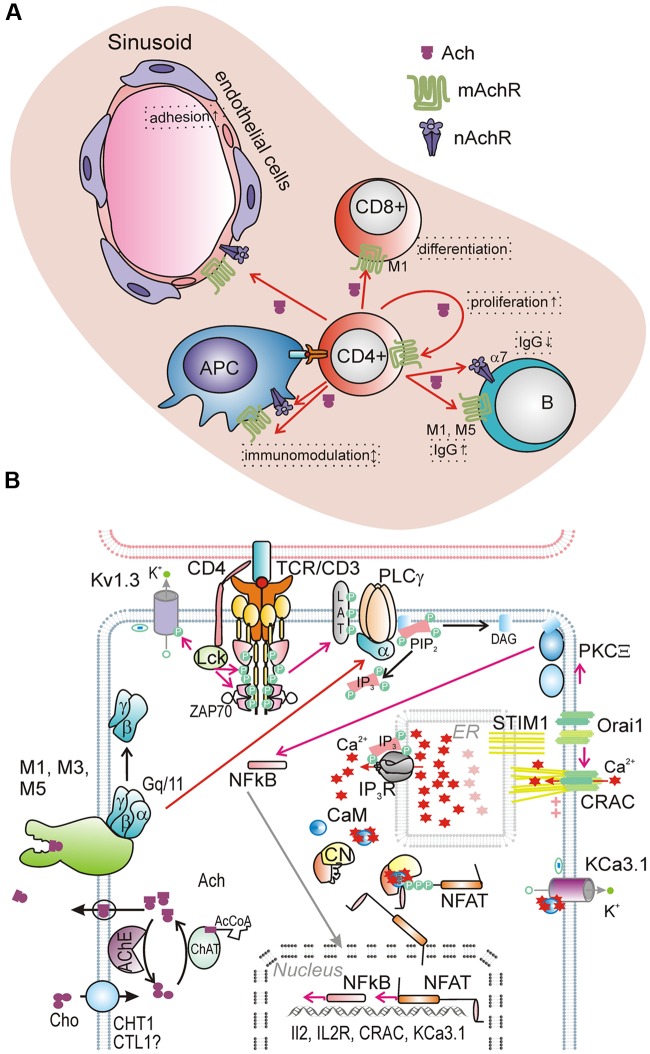
**Current view on T cell NNCS in regulation of immune function.** **(A)** Proposed paracrine and autocrine effect of T cell-derived ACh in T cell microenvironment. **(B)** Role of NNCS in Ca^2+^ signaling and gene expression in healthy T-lymphocyte. **(A)** TCR- and muscarinic (M1, 3, 5) receptor- triggered signaling pathways are cross-linked in T cells. In healthy T-cells, principle route of phospholipase C (PLCγ) activation upon recognition of antigen-presenting cell occurs via TCR/CD3 complex. TCR –generated signal is amplified by a co-receptor (CD4), which recruits Lck, a tyrosine kinase, phosphorylating several targets, including Z-chain protein kinase ZAP70, which phosphorylates LAT (linker for activation of T-cells), interacting with PLCγ. PLCγ may be activated through alternative mechanism related to T cell cholinergic system, where import of choline (Cho), synthesis of ACh and its secretion via yet unknown pathway results in paracrine/ autocrine activation of muscarinic receptors M1, M3 and/or M5. It follows by activation of trimeric G-protein (G_q_11), whose subunit α activates PLCγ. PLCγ degrades phosphatidylinositol 4,5-bisphosphate (PIP_2_) into soluble IP_3_ and membrane bound diacylglycerol (DAG). Intracellular release of IP_3_ activates IP_3_ receptor-channel, mediating Ca^2+^-release from ER. Ca^2+^ depletion of reticulum is sensed by STIM1 of ER membrane, which promotes assembly of Orai subunits and formation of CRAC channel in the plasma membrane, the main route of capacitive Ca^2+^ entry, which considered to be principle Ca^2+^ influx channel in lymphocytes. CRAC-mediated Ca^2+^ influx is sustained, when membrane negative potential difference is maintained due to K^+^ efflux via a set of K^+^-selective channels, in healthy T-cells represented by voltage-dependent K_v_1.3 channel and Ca^2+^-activated intermediate conductance K^+^ channel KCa3.1. Activation of calcineurin (CN) via Ca^2+^/calmodulin (CaM) complex occurs only upon prolonged intracellular Ca^2+^ increase. Principle target of CN is NFATs, heavily phosphorylated at the rest; its dephosphorylation make it permeable via nuclear pore. By entering nucleus, NFAT induces the transcription of several genes.

**Figure 1A** depicts our current understanding of possible cellular targets for the T-cell derived ACh. However, it must be kept in mind that various cell lineages from CD4^+^ cell microenvironment may also produce ACh and that real interactions are more complex.

### Acetylcholine-Producing T Cells are Key Regulators in the Cholinergic Anti-inflammatory Pathway

The cholinergic anti-inflammatory pathway, also termed as inflammatory reflex, together with anti-inflammatory cytokines, glucocorticoids, and other humoral mediators, is involved in the suppression of inflammatory response (Tracey, 2002). An excellent critical review highlighting the evolution in the understanding of underlying mechanisms was published recently (Martelli et al., 2014). Here, we only briefly present this well-studied example, to emphasize the potential of the T cell NNCS in mammal physiology.

During the inflammation, pro-inflammatory cytokines activate afferent sensory neurons, which represent the sensory arc of the inflammatory reflex. Axons traveling in the vagus nerve transmit this information as action potentials to the brain stem. The brain stem generates inhibitory signals, which traverse the efferent vagus nerve into the spleen. This efferent arc actually represents a cholinergic anti-inflammatory pathway. Electrical stimulation of splenic nerve was believed to result in release of ACh into splenic tissue and into the effluent splenic vein, with a subsequent down regulation of the inflammatory cytokines (Tracey, 2009). Rosas-Ballina et al. (2011) showed that the ACh released in response to vagal stimulation was not neural in its origin. Using a murine model, they demonstrated that ACh was produced by a subset of T lymphocytes, present in the spleen (Rosas-Ballina et al., 2011; Gautron et al., 2013). Consequently, in nude mice, lacking functional T cells, vagal stimulation had no anti-inflammatory effect, whereas it was restored by the adoptive transfer of ACh-synthesizing T lymphocytes (Rosas-Ballina et al., 2011). Direct binding of the ACh to α7 nAChR on macrophages was proposed (Rosas-Ballina et al., 2011). However, according to a more recent model, ACh liberated from mobilized T cells binds primarily to the essential α7 nAChR on the peripheral terminals of the splenic sympathetic nerves and stimulates noradrenaline release (Martelli et al., 2014). Noradrenaline in turn acts on β-adrenergic receptors on splenic macrophages, with a resulting suppression of the TNF-α production. Direct binding of ACh to α7 nAChR on macrophages is also possible (Rosas-Ballina et al., 2011; Martelli et al., 2014). In this way, the production of ACh by T cells provides a reliable protective mechanism from inflammatory damage in mammals.

### TCR- and Muscarinic (M1, 3, 5) Receptor- Triggered Signaling Pathways are Cross-Linked in T Cells

Mature naïve or memory T cells are activated following recognition and binding of TCR to a specific antigen, presented by infected or professional antigen-presented cells. This initial event triggers the signaling cascade, which includes activation of the PLCγ, hydrolysis of phosphatidylinositol 4,5-bisphosphate (PIP_2_) with the release of IP_3_, IP_3_ binding to its receptor in the ER, initial Ca^2+^ release from ER with a subsequent activation of CRAC of the cell membrane and a complex modulation of Ca^2+^ signaling, leading to the Cn activation. NFATs is a principal Cn target in T lymphocytes. NFAT dephosphorylation and translocation to nucleus as well as the activation of PKC and MAPK cascades culminate in changes of the transcriptional activity and proliferation of an antigen-specific clone (Lichtman et al., 1983; Weiss and Littman, 1994).

Among genes up-regulated during T cell activation, IL-2 and IL-2R are of special importance for a clonal expansion. Signaling through TCR/CD3 stimulates both ACh production and its release from T cells, as demonstrated in experiments with T cells, treated with PHA, which crosslinks TCR (Fujii et al., 1998; Kawashima and Fujii, 2000; Fujii et al., 2012b). In turn, lymphocyte-derived ACh acts in an autocrine and/or paracrine manner, via binding to the AChR on the cellular surface. Muscarinic receptors M1, M3, and M5 are coupled to Gq/11 type G proteins and activate PLC, which releases IP_3_. In a variety of experimental systems, cholinergic agonists caused intracellular Ca^2+^ rise and c-fos expression (Fujii and Kawashima, 2000a,b,c; Kawashima and Fujii, 2003) and led to the expression of both IL-2 and IL-2R in T lymphocytes (Rinner et al., 1995; Kawashima and Fujii, 2000, 2003; Nomura et al., 2003). Activation of these receptors has been linked to the control of MAPK and, as a consequence, to the regulation of cell growth and proliferation (Gutkind, 1998; Gudermann et al., 2000).

Accordingly, signaling events of the both mentioned pathways appear to be cross-linked, with the autocrine stimulation by ACh creating an amplifying loop in the TCR signaling (**Figure 1B**).

## Cholinergic Signaling May Be Involved in T Leukemogenesis

T leukemogenesis is a multistep process, where genetic aberrations during the T cell maturation convert healthy T cell progenitors into leukemic cells that are unable to differentiate, but demonstrate high potential of self-renewal and proliferation. Due to multiple mutations, T-ALL are heterogeneous and multiclonal. Molecular basis of T-ALL pathogenesis is a subject of intensive studies and is reviewed exhaustively elsewhere (Graux et al., 2006; Van Vlierberghe and Ferrando, 2012; Hales et al., 2014; Durinck et al., 2015). Among most frequent and prominent abnormalities are activating mutations in NOTCH1, IL7R/JAK, and PI3K/Akt/mTOR pathways. The majority of signaling pathways, up-regulated by leukemogenic mutations, strongly depend on Ca^2+^ signaling (reviewed in Dobrovinskaya et al., 2015). For example, the NOTCH1- dependent Cn–NFAT axis, aberrantly activated in over 50% of pediatric T-ALL (Weng et al., 2004), requires a sustained elevation of intracellular Ca^2+^ (Medyouf et al., 2007; Gachet et al., 2013).

As described in previous chapters, the cholinergic system is involved in the regulation of Ca^2+^ signaling in lymphocytes. Importantly, T cell progenitors show the presence of a cholinergic autocrine/paracrine loop from the earliest stages of maturation (Rinner et al., 1999). This mechanism may be retained in aberrant leukemic progenitors, and involved in the maintenance of an increased proliferation level. In this chapter we will first provide a summary on the expression and function of cholinergic elements in tumors of different origin, and then present the data, available for T-ALL. We will also discuss a possible relation between genetic aberrations and cholinergic machinery during T leukemogenesis. Special emphasis will be put on a potential role of the cholinergic autocrine/paracrine loop in a shaping of Ca^2+^ signal and on the involvement of cholinergic signaling in interactions between leukemic T cells and their microenvironment in the BM.

### Cholinergic Signaling in Cancer

An enhanced level of choline uptake and metabolism is obviously required for actively proliferating tissues, because choline is needed for a rapid biosynthesis of cell membranes. Elevated concentrations of choline and its metabolites were revealed by means of MRS in a wide variety of malignances (reviewed in Inazu, 2014). Accelerated choline metabolism was considered as a metabolic hallmark that is associated with malignant progression (reviewed in Glunde et al., 2011). Nowadays, MRS method which determines elevated local concentrations of choline and its metabolites is widely used to assist the diagnosis and staging of cancer, and evaluate the therapeutic response (Hara et al., 1997, 1998, 2000; Glunde et al., 2006; García-Figueiras et al., 2016).

At the same time, choline is needed for the ACh synthesis, which acts as an autocrine/paracrine growth factor in various types of healthy and tumor cells, thus regulating their proliferation. Several excellent reviews regarding involvement of NNCS in tumorigenesis have been published recently (Spindel, 2012; Russo et al., 2014; Campoy et al., 2016). In particular, lung, melanoma, pancreas, breast, and colon cancers represent examples, where more details related to cholinergic autocrine/paracrine loop are available. Detailed analysis of the expression of NNCS elements in tumors of different histogenesis revealed that they largely reflected their expression in the original normal tissue (Spindel, 2012). At the same time, the ACh synthesis was often enhanced in tumors, and some differences in AChR expression patterns were observed. In general, α7-nAChR and M3 mAChR were shown to be the most capable among AChR to promote cell proliferation and cancer progression. On the other hand, expression of the α9-nAChR was also described in breast cancer cells; α5-, β2- and β4-nAChR – in broncho-pulmonary carcinomas; α3- and α4-nAChR – in pancreatic ductal adenocarcinoma (reviewed in Russo et al., 2014; Campoy et al., 2016; Dang et al., 2016).

The involvement of M3 mAChR in cancer progression was related to autocrine/paracrine ACh production. A variety of cell types in normal lungs synthesizes and releases ACh, which acts as a growth factor (Reinheimer et al., 1996). However, the same cholinergic loop may be also involved in the lung cancers progression (Song et al., 2003). Indeed, as it was shown by Song et al. (2003), SCLC cell lines express ChAT, VAChT, CHT1, and both nAChR and mAChR genes, and they synthesize, secrete and degrade ACh. Positive ChAT immunostaining was present not only in SCLC cell lines, but also in biopsies, derived from the SCLC. Muscarinic receptor agonists caused a concentration-dependent intracellular Ca^2+^ rise, which, in turn, leads to the activation of Akt and MAPK. This effect was canceled by subtype-selective mAChR antagonists and siRNA. Multiple M3 antagonists, including 4-diphenyl-acetoxy-N-methyl-piperidine (4-DAMP), P-F-HHSiD, darifenacin, and tiotropium were shown to inhibit lung cancer cell proliferation *in vitro*, and darifenacin and tiotropium were effective in inhibiting lung cancer growth *in vivo* in nude xenograft mice. Thus, among AChR, M3 receptors were suggested as the most important in the development of lung cancer (Song et al., 2003, 2007, 2008, 2010). As was reported earlier, M3 can regulate the activity of E-cadherin and integrins, which are considered as key factors in cell adhesion and migration (Williams et al., 1993). In keeping with those findings, it was demonstrated recently that the M3 AChR promoted invasion and migration of NSCLC cells through the Akt-dependent pathway (Lin et al., 2014). The same authors reported that overexpression of the M3 receptor promotes metastasis and predicts poor prognosis in NSCLC patients (Lin et al., 2014). Together with an auto-activation mechanism, dependent on ACh secretion, the involvement of constitutive ligand-independent receptor activity was also proposed (Spindel, 2012).

Predominant M3 expression was also demonstrated in melanoma (Lammerding-Köppel et al., 1997; Boss et al., 2005; Oppitz et al., 2008), although normal melanocytes were reported to express all M2–M5 receptors (Buchli et al., 2001). Moreover, M3 receptors expression is elevated in leading edges of tumors and in metastases (Lammerding-Köppel et al., 1997; Oppitz et al., 2008). In accordance with this, it was proposed that the M3 receptors play a pivotal role in the chemotaxis of melanoma cells (Boss et al., 2005).

There is abundant evidence for a predominant role of M3 receptors in colon cancer. Frucht et al. (1999) demonstrated that the M3 expression was increased about eight-fold in tumor versus normal tissue, and that the cholinergic agonist carbamylcholine stimulated the proliferation of M3-expressing colon carcinoma cell lines (Yang and Frucht, 2000). These findings were confirmed by other research groups, who demonstrated that the proliferative action of the M3 receptors depended partially on the *trans-*activation of epithelial growth factor (EGF) receptors (Raufman et al., 2003; Ukegawa et al., 2003). Later, it was shown that the M3 receptor knockout mice were resistant to the development of colon tumors in the azoxymethane-induced colon neoplasia model (Raufman et al., 2008).

Tumors of different histogenesis also express nAChR, and a possible role of nAChR in tumor progression was also reported (reviewed in Russo et al., 2014; Dang et al., 2016; Zhao, 2016). Since mAChR demonstrate the higher affinity for ACh than nAChR, the ACh produced by tumors is likely to act primarily through muscarinic pathways. The situation may change if some additional factors, like chronic nicotine consumption, significantly increase the expression of nAChR. Indeed, positive correlations between nAChR signaling and cigarette smoking were revealed for lung, pancreatic, and esophageal cancers. Possible mechanism depicting how chronic nicotine exposure may affect the profile of nAChR expression in affected tissues, was proposed recently (Schuller, 2009; Zhao, 2016). α7-nAChR were suggested to be the most growth stimulatory, whereas α4β-nAChR are growth inhibitory. Normally, nicotine binds to α4β-nAChR with a higher affinity than to α7-nAChR. But chronic exposure to nicotine or nicotine-derived nitrosamines leads to inactivation (desensitization) of the α4β-nAChR and simultaneous upregulation of α7-nAChR, wherein the sensitivity of the latter to nicotine remains unchanged. α7-nAChR are highly selective for Ca^2+^ when compared to others nAChR, and Ca^2+^ influx seems to be related to the activation of Akt and MAPK signaling pathways. Besides, Ca^2+^ influx triggers plasma membrane depolarization, which activates voltage-gated Ca^2+^ channels (but not in lymphocytes, where they are functionally inactive) and consequently prolongs Ca^2+^ influx. Interestingly, proliferative effect of nicotine may be abolished by darifenacine, an M3-selective antagonist, indicating that a cross-talk between signaling pathways may occur. Additionally, human-specific dupα7-nAChR protein could modify the function and ligand tropism of the normal α7-nAChR ligand-gated channel. However, expression levels of the corresponding CHRFAM7A gene in tumors were not evaluated yet.

### Muscarinic Receptors and Drug Resistance

Gq/11-coupled subtypes of mAChR (M1, M3, and M5) are able to protect against the apoptotic cell death caused by drug treatment. CHO cells, which stably express these, but not M2 or M4 receptors, demonstrate a significant inhibition of the etoposide-mediated caspase-3 activity, phosphatidylserine externalization, and DNA degradation in the presence of the cholinergic agonist carbachol. Surprisingly, the protective effect was not related to the PLC pathway. Instead, the conserved poly basic region in the C-terminal tail of the M1, M3, and M5 receptors contributes to the ability of these receptors to mediate a protection against apoptosis (Budd et al., 2003; Tobin and Budd, 2003).

### Cholinergic Elements in Leukemic T Cells

Numerous lines of evidence exist, demonstrating that healthy CD4^+^ T cells possess a cholinergic autocrine loop which regulates their proliferation. As leukemic T cells often display an enhanced ACh production and express various AChR, there is even more reason to suggest an involvement of similar mechanism in pathogenesis of T-ALL, especially bearing in mind the establishment of such a mechanism for other tumor types.

Acetylcholine production by T cell lines derived from leukemic patients (CEM, HSB-2, Jurkat, MOLT-3, and MOLT-4) is considerably higher when compared to unstimulated MNL, obtained from healthy donors (Fujii et al., 1996; reviewed in Kawashima and Fujii, 2004). The highest ACh content was found for MOLT-3 cell line, derived from a patient in the relapse phase after the multidrug chemotherapy. It appears not to be coincidental that MOLT-3 also expresses the CHT1, which could mediate an efficient uptake of choline needed for the ACh synthesis. Considering a TCR-independent proliferation of leukemic cells and the cross-talk between TCR- and mAChR-triggering signaling pathways, it is tempting to suggest that ACh, which is produced in large quantities, may act as an autocrine signal molecule and contribute to the clonal expansion in T-ALL. Moreover, ACh produced by T-ALL is not rapidly hydrolyzed, because AChE activity is drastically decreased in these cells, in contrast to mature T lymphocytes (Rubinstein et al., 1984). On the other hand, an increased AChE activity was reported in total blood and lymphocytes samples, obtained from pediatric patients, newly diagnosed with T- or B-ALL, whereas it decreased during the remission period. It could be suggested that the elevated AChE activity is related to the enhanced ACh content and represents an adaptation mechanism, but the origin of this additional AChE activity remains unknown (Battisti et al., 2009).

Presence of the mRNA encoding nicotinic α3, 5, 6, 9, and 10 subunits was reported in acute T-leukemic cell lines CEM and Jurkat (Peng et al., 2004; Landais et al., 2010). Jurkat, but not CEM, also expressed α7 (Landais et al., 2010). Comparative analysis of mAChR expression was undertaken in Jurkat, a very popular model for studies of the T cell signaling pathways. The transcriptional profile of mRNA coding for various mAChR isoforms in Jurkat cells demonstrated a robust M3 expression, which was approximately threefold higher than that of both M5 and M4, whereas M1 and M2 were undetectable (Alea et al., 2011). This result agrees with earlier study where expression of the M3 in Jurkat was evidenced by Northern blot (Kaneda et al., 1993). In the same work (Kaneda et al., 1993), the effect of several cholinergic agonists on intracellular Ca^2+^ level in Jurkat cells was studied. Nicotine had no effect at all indicating that nAChR seem not to form functional channels in these cells. On the other hand, ACh, carbachol, muscarine and M1/M3 agonist Oxo-M induced transient Ca^2+^ rise, wherein Oxo-M caused the greatest effect. McN-A343 (a selective M1 agonist), pilocarpine (potent M1 agonist) and oxotrimarine (a potent M2 agonist) had no or only slight effect. Differential inhibitory analyses of Oxo-M-induced Ca^2+^ rise with muscarinic antagonists revealed a functional involvement of the M3 receptor. Transient elevation of the intracellular Ca^2+^ was followed by the activation of PLC and IP_3_ formation. These data undoubtedly evidenced a specific importance of M3 receptor in the cholinergic loop of Jurkat leukemic cells.

More recently, two preliminary studies have reported that nicotine may still induce α –BTX- sensitive Ca^2+^ increase in CEM or Jurkat leukemic T cell lines (Kimura et al., 2003; Landais et al., 2010). It should be noted that in the first paper only 15% of CEM cells were shown to respond to nicotine, whereas in the second one no Ca^2+^ response was detected in CEM cells, albeit extremely high (10 mM) nicotine concentration was utilized. A simple mechanism, where nicotine-induced Ca^2+^ rise in T lymphocytes is mediated by Ca^2+^ influx via a plasma membrane nAChR was questioned in a more detailed study on Jurkat cells (Razani-Boroujerdi et al., 2007). Nicotine-induced Ca^2+^ increase in these experiments was shown to be independent on external Ca^2+^. Hence, it is caused by intracellular Ca^2+^ release and not by a Ca^2+^ influx via nAChR, which appear not to form functional channels in plasma membrane of T cells (Cui and Li, 2010). Moreover, α -BTX and methyllycaconitine, two relatively specific antagonists of α7, α9, and α10 nAChR, instead of inhibition of the nicotine induced Ca^2+^ rise, by themselves cause cytosolic Ca^2+^ increase similar to that induced by nicotine (Razani-Boroujerdi et al., 2007). Thus, unexpectedly, these compounds hit alternative molecular targets in T cells and their use as functional probes for an active nAChR may be challenged in this case. Also, intracellular Ca^2+^ increase reported in the last study was relatively low (less than 150 nM) and its kinetics showed significant variations from sample to sample (Razani-Boroujerdi et al., 2007). Collectively, the above mentioned studies pointed to a limiting role of nAChRs in Ca^2+^ signaling in leukemic T cells. In the human leukemic T cell line CEM, nicotine (0.01–10 μM) down-regulates mRNA expression for all the nAChR subunits (reviewed in Kawashima et al., 2012).

### Are Cholinergic Pathways Involved in the Interaction of Leukemic T Cells with their Microenvironment in Bone Marrow?

**FIGURE 2:**
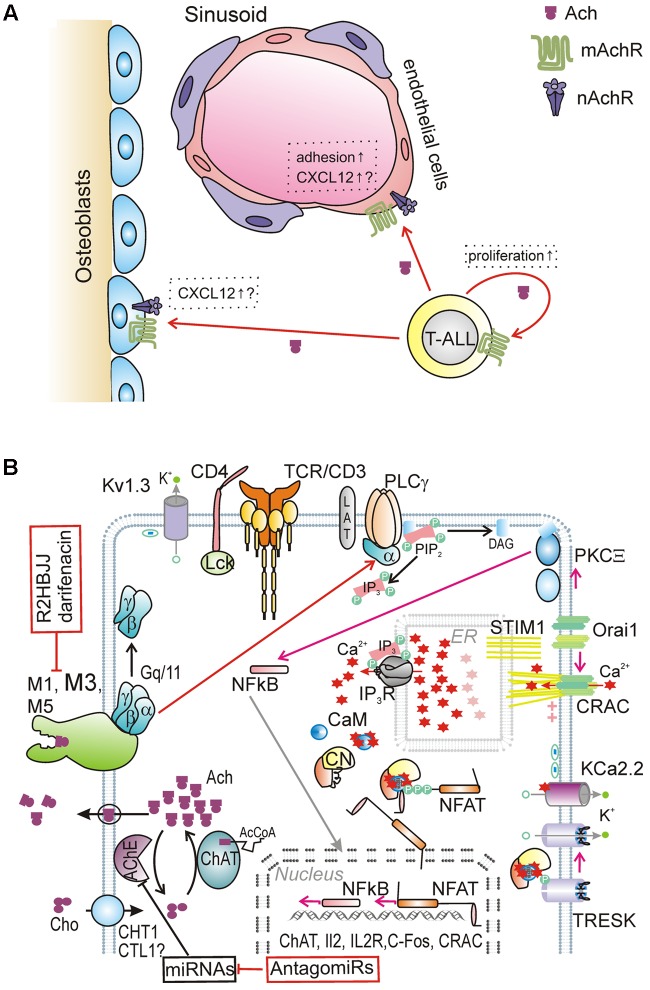
**Hypothetical model of NNCS involvement in T leukemogenesis.** **(A)** Autocrine and paracrine effect of ACh derived from T leukemic cell in specific leukemic niche in BM. **(B)** Network of signaling events in leukemic T lymphoblasts. It is suggested, that in absence of TCR/CD3 stimulation in leukemic T cells, elevated level of ACh production and autostimulation through M (1, 3, 5) receptors may play key role in signaling network. The sustained CRAC-mediated Ca^2+^ influx is maintained due to K^+^ efflux via a set of K^+^-selective channels: voltage-dependent K_v_1.3 and small conductance KCa2.2; TWIK-related tandem pore K^+^ channel TRESK may be also recruited being activated by dephosphorylation by CN. Other abbreviations: NFkB (nuclear factor kappa-light-chain-enhancer of activated B cells), PKC (protein kinase C), IL (interleukin), C-Fos (proto-oncogene, transcription factor), CHT1 (high-affinity choline transporter 1), CTL1-5 (intermediate-affinity choline-transporter-like proteins), ChAT (choline acetyltransferase), AcCoA (acetyl coenzyme A).

The BM microenvironment is known to regulate the properties of healthy hematopoietic stem cells localized in specific niches. Two distinct microenvironmental niches, namely “osteoblastic (endosteal)” and “vascular,” have been identified in the BM. It was proposed recently that in leukemic individuals these niches represent specific places, where subsets of leukemic cells escape the chemotherapy-induced death and acquire a drug-resistant phenotype. Moreover, there is emerging evidence that leukemia cells are able to remodel the BM niches into malignant ones, which may better support neoplastic cell survival and proliferation (Chiarini et al., 2016). T-ALL cells were demonstrated to be in a direct stable contact with BM stromal cells, namely vascular endothelium and osteoblasts, producing chemokine CXCL12, an important component for the T-ALL pathogenesis; CXCL12 acts via the CXCR4 receptor, which is expressed in leukemic cells (Pitt et al., 2015; Passaro et al., 2016). As T-ALL produce a large quantity of ACh, it may exert a significant paracrine effect on neighboring stromal cells. Indeed, both osteoblasts and endothelial cells were reported to express both nAChR and mAChR (Sato et al., 2010; Behringer and Segal, 2015). As a model, ACh produced by leukemic T cells may interact with corresponding receptors on stromal cells and play an important role in the remodeling of microenvironmental niches (**Figure 2A**).

### Genetic Aberration in T Leukemias: Are They Related to the Cholinergic Machinery?

T leukemogenesis is a multistep process, with different genetic aberrations interacting in progenitor T cells, to produce leukemogenic phenotype characterized by differentiation arrest and enhanced self-renewal and proliferation capacity. Up-regulation of transcription factors TLX1 (HOX11), TLX3 (HOX11L2), and SIL/TAL1 are the most frequent changes, where the TLX3 expression was related to an unfavorable outcome (Graux et al., 2006). For the moment, the data available about possible involvement of these factors in the up-regulation of cholinergic system are limited. Yet, noteworthy, the expression of transcription factor TLX3 in prenatal sympathetic neurons during embryonic development is restricted to the cholinergic population and the TLX3 deletion results in a loss of some cholinergic components in these cells (Furlan et al., 2013; Huang et al., 2013). Thus, TLX3 is apparently required for the acquisition of a cholinergic phenotype at the late embryonic stage as well as throughout the prenatal development in mouse sympathetic neurons. TLX3 repression resulted in the development of a noradrenergic, instead of cholinergic phenotype (Furlan et al., 2013). Among 2462 genes, putative TLX3 targets, BCHE (coding pseudocholinesterase) and CHRFAM7A (coding dupα7), can be found (Pujato et al., 2014). Whereas, dupα7 is a dominant-negative regulator of the α7 ion channel function, these data argue that the expression of functional α7 nAChR may be decreased in T-ALL with TLX3 up-regulation.

### Cholinomirs: A Novel Approach for Leukemias?

MicroRNAs (miRNAs) are short single-stranded non-coding RNAs that influence post-transcriptional gene regulation by affecting the mRNA stability and/or translational repression of their target mRNAs. They are involved in several pathophysiological processes, including differentiation, proliferation, apoptosis, metabolism, hematopoiesis, immune response, and cancer (Ha, 2011; Liu and Abraham, 2013). Furthermore, miRNAs are associated with the regulation of the cholinergic system (Priyadarshini et al., 2013).

Accordingly, miR-132 and miR-186 down-regulate AChE through mRNA-targeting (Shaked et al., 2009; Maharshak et al., 2013; Nadorp and Soreq, 2014). In addition, miR-124 mediates the cholinergic anti-inflammatory action, by inhibiting the pro-inflammatory cytokines production (Sun et al., 2013). These miRNAs have been designated as “CholinomiRs,” for being involved in the cholinergic signaling (Nadorp and Soreq, 2014).

On the other hand, the participation of miRNAs in cancer development is multifaceted, given that they play a role as tumor suppressors or tumor promoters, depending on their particular targets (Chen et al., 2012). Previous reports suggested that differential miRNA expression has a potential role in leukemias and is associated with drug resistance, disease progression and prognosis (Schotte et al., 2012; Bottoni and Calin, 2014). Recently, an *in silico* analysis predicted a group of miRNAs that is able to regulate the AChE expression (Hanin and Soreq, 2011; Nadorp and Soreq, 2014). Comparative analysis of these miRNAs with the miRNAs in acute leukemia of different cytogenetic and molecular subtypes identified the following “CholinomiRs”: miRs -125b, -196a, -320, -708 in children, and miRs-24, -125b, -181a-d, and miR-196a in adults (Schotte et al., 2012). Additionally, the up-regulation of miR-132 in chronic lymphocytic leukemia was shown to be related to disease progression (Tavolaro et al., 2015) and the decrease of miR-124 predicted a favorable prognosis in acute myeloid leukemia (Chen et al., 2014). For these reasons, “CholinomiRs,” implicated in the T leukemia pathogenesis, may be attractive targets for future therapeutic strategies.

### Cholinergic System may be Involved in Shaping of Ca^2+^ Signals in T Leukemias

As it was emphasized in previous chapters, cholinergic and TCR-triggered signaling pathways interconnect at the point of the PLC activation, PIP_2_ hydrolysis, and generation of the two second messengers, IP_3_ and diacylglycerol (DAG). Whereas DAG activates the RAS/PKC pathway, IP_3_ induces the release of Ca^2+^ from the ER by activating the IP_3_R Ca^2+^-permeable channel. Strikingly, Ca^2+^ release from intracellular stores, independent of extracellular Ca^2+^, also takes place upon the stimulation of T- (here, Jurkat) cells by nicotine. nAChR (here, the α7 isoform) does not form a functional Ca^2+^-permeable channel, but acts in an allosteric manner in concert with a functional TCR and leukocyte-specific tyrosine kinase (Razani-Boroujerdi et al., 2007).

Depletion of ER Ca^2+^ stores is sensed by the STIM1 protein, integrated in the ER membrane, which in turn induces the opening of ORAI1 (CRAC) channels, located in the plasma membrane and mediating Ca^2+^ influx. To maintain Ca^2+^ influx through CRAC, the principal Ca^2+^-selective channel in T cells, which mediates a current with a substantial inward rectification, membrane potential needs to be kept negative (hyperpolarized). Membrane hyperpolarization may be achieved via the activity of potassium channels. Voltage-dependent Kv1.3 and especially (due to their relatively high activity at hyperpolarized potentials) Ca^2+^-activated KCa3.1 channels are involved (Cahalan and Chandy, 2009; Feske et al., 2015). Maintenance of negative membrane potential is also essential for choline uptake by CHT1, due to a voltage-dependence of the latter (Iwamoto et al., 2006), which in turn may control the ACh biosynthesis rate.

In contrast to healthy T cells activated by antigens, clonal expansion of T leukemic cells is TCR-independent. Nevertheless, constitutively high levels of Cn activity have been detected in leukemic cells. As it was mentioned, we suggest that an elevated ACh production with consequent autocrine stimulation through AChR may contribute significantly to this process (**Figure 2B**). But what about K^+^ channels, which support, via membrane hyperpolarization, a sustained Ca^2+^ influx, required for the Cn activation? Acute T-ALL Jurkat cell line displays several-fold lower Kv1.3 channels density; it was reported also that these cells express small conductance KCa2.2 channels in increased numbers, compared to the levels of KCa3.1 channels in activated healthy T cells (reviewed in Dobrovinskaya et al., 2015). Data from our lab demonstrated that Jurkat cells and other T-leukemic cell lines as well as blood samples from patients with T-ALL show the expression of a tandem-pore K^+^ (K2P) channel TRESK (Pottosin et al., 2008; Sánchez-Miguel et al., 2013). Data from other group also revealed the presence of the members of K2P channels in human T-cells (Meuth et al., 2008; Andronic et al., 2013).

Noteworthy, TRESK is the only member of the K2P family which is activated, albeit indirectly, by elevated intracellular Ca^2+^, due to the dephosphorylation of several serine residues in its NFAT -like domain by Cn (Czirják et al., 2004; Han and Kang, 2009; Enyedi and Czirják, 2014). Thus, TRESK is another target for Cn in leukemic T-cells. In contrast to Ca^2+^-activated channels KCa3.1 and KCa2.2, whose activities are ceased immediately after Ca^2+^ removal, TRESK stays active until it is rephosphorylated. Thus, it could support a more robust and sustained Ca^2+^ signal, via a positive feedback loop as TRESK and CRAC may support each other’s activity mutually: TRESK activation by Ca^2+^- Cn and CRAC activation by TRESK-mediated hyperpolarization. Importantly, TRESK activity could be evoked via G protein-coupled receptor pathway, by activating muscarinic receptors or inducing Ca^2+^ release from the ER by IP_3_ (Czirják et al., 2004). To summarize, in leukemic T-cells TRESK could be directly involved into gene expression regulation by NFAT, as both TRESK and NFAT are activated in the same manner via Ca^2+^-dependent dephosphorylation by Cn. Positive feedback between TRESK and intracellular Ca^2+^ increase may be further re-inforced due to the elevated ACh production and autocrine stimulation through the mAChR ligation (Dobrovinskaya et al., 2015).

## Outlook

Dysregulation in function and expression of the NNCS components were reported to be important factors in cause and progression of several types of cancers. Therefore, pharmacological agents interfering with the NNCS were suggested to be very promising for the anti-cancer therapy (Beckmann and Lips, 2013). Evidently, more research is required to completely elucidate the function of the NNCS in normal physiology of different tissues and in pathological states. At the same time, current data may already point to suitable molecular targets for new therapeutic strategies.

ACh produced by T cell in a healthy organism plays a key role in the anti-inflammatory pathway (inflammatory reflex). Furthermore, the autocrine cholinergic loop represents an important regulatory mechanism during maturation, activation and proliferation of T cells. Noteworthy, T leukemic cell lines derived from relapsed T-ALL patients produce significantly higher levels of ACh in comparison to healthy T cells. In the absence of TCR-dependent signaling in leukemic cells, elevated quantities of ACh, as autocrine/paracrine regulator, may play an important role in shaping of Ca^2+^ signals and in clonal expansion. Similar to other types of cancers, some T-cell derived leukemic lines demonstrated the up-regulation of M3 receptor. Transcription factor TLX3, up-regulated in more than 50% of T-ALL (Graux et al., 2006), is crucial for acquisition of cholinergic machinery (Furlan et al., 2013; Huang et al., 2013). Altogether, these data indicate that the NNCS is involved in T leukemogenesis and that its elements may represent a relevant therapeutic target.

Although, muscarinic antagonists have not been used yet in clinical practice as anti-cancer drugs, this therapeutic strategy is considered and has been discussed for future trials. Taking into account the evidence about a predominant role of M3 receptors in growth of different types of tumors, widespread clinical use and low toxicity of M3 antagonists, a potential role of these drugs as adjuvants for cancer therapies looks especially attractive (reviewed in Spindel, 2012). As was reported recently, several muscarinic antagonists, already used in the treatment of genitourinary diseases (e.g., darifenacin, patents US5096890 and US6106864), have also been demonstrated to arrest the tumor growth *in vivo* (Matera and Tata, 2014).

Promising results were also obtained with a new anticholinergic agent R2HBJJ which has a high affinity for M1 and M3 AChR. R2HBJJ was designed for the treatment of peripheral diseases with reduced access to the central nervous system. It inhibited NSCLC growth by a mechanism, involving down-regulation of cyclin D1-CDK4/6-Rb and arrests the cell cycle in G0/G1 (Hua et al., 2012).

Taking into account these findings, it would be of special advantage to reveal the effect of muscarinic antagonists on proliferation and clonal expansion of leukemic cells, with subsequent study of underling molecular mechanisms. Another strategy may involve up-regulation of AChE through miRNAs-dependent mechanism (AntagomiRs), with a resulting increase of the ACh degradation in the leukemic cells (**Figure 2B**). Rather than being used as alternatives, these treatments should be combined with conventional anti-cancer therapies, in order to develop flexible multi-drug protocols to combat T-ALL, a rather heterogeneous and multifaceted disease.

## Author Contributions

OD: generated an original idea, made a plan and index, distributed chapters between the authors, performed database search, wrote several chapters, performed final editing, writing, editing, and proof-reading. GV-C: performed database search, contributed in chapter writing and made a proof reading. LC-S: performed database search, wrote two chapters, critically discussed the content of the final version. EB-A: performed database search and wrote a chapter. LL-R: performed database search and wrote a chapter. IP: performed database search, wrote a chapter and designed the figures.

## Conflict of Interest Statement

The authors declare that the research was conducted in the absence of any commercial or financial relationships that could be construed as a potential conflict of interest.

## Abbreviations

4-DAMP: 4-diphenyl acetoxy-*N*-methyl-piperidine
ACh: acetylcholine
AChE: acetylcholinesterase
AChR: ACh receptors(s)
ALL: acute lymphoblastic leukemia
B-ALL: ALL of B cell lineage
BChE: butyrylcholinesterase
BM: bone marrow
Ca^2+^: calcium
ChAT: choline acetyltransferase
CHT1: high-affinity choline transporter 1
Cn: calcineurin
CTL1-5: intermediate-affinity choline transporter-like proteins
CRAC: Ca^2+^ release activated channels
DCs: dendritic cells
ER: endoplasmic reticulum
IL-2: interleukin-2
IL-2R: IL-2 receptor(s)
IP_3_: inositol-1,4,5-trisphosphate
mAChR: muscarinic ACh receptor(s)
MAPK: mitogen-activated protein kinase
MicroRNAs: miRNAs
MNLs: mononuclear leukocytes
MRS: magnetic resonance spectroscopy
nAChR: nicotinic ACh receptor(s)
NFATs: nuclear factor of activated T cells
NNCS: non-neuronal cholinergic system
NSCLC: non-small-cell lung cancer
OCT: organic cation transporter
P-F-402: HHSiD parafluorohexahydrosiladifenidol
PHA: phytohemagglutinin
PKA: protein kinase A
PKC: protein kinase C
PLC: phospholipase C
PMA: phorbol 12-myristate acetate
SCLC: small cell lung carcinoma
STIM1: stromal interaction molecule protein 1
T-ALL: ALL of T cell lineage
TCR: T cell receptor(s)
VAChT: vesicular ACh transporter
